# Population Structure and Genetic Diversity Analysis of “Yufen 1” H Line Chickens Using Whole-Genome Resequencing

**DOI:** 10.3390/life13030793

**Published:** 2023-03-15

**Authors:** Cong Liu, Dongxue Wang, Yuehua He, Wenjie Liang, Wenting Li, Kejun Wang, Donghua Li, Zhuanjian Li, Yadong Tian, Xiangtao Kang, Guirong Sun

**Affiliations:** 1 College of Animal Science and Technology, Henan Agricultural University, Zhengzhou 450001, China; 2 Henan Key Laboratory for Innovation and Utilization of Chicken Germplasm Resources, Henan Agricultural University, Zhengzhou 450001, China; 3 The Shennong Laboratory, Zhengzhou 450002, China

**Keywords:** “Yufen 1” H line, genetic diversity, population structure, whole-genome resequencing

## Abstract

The effective protection and utilization of poultry resources depend on an accurate understanding of the genetic diversity and population structure. The breeding of the specialized poultry lineage “Yufen 1”, with its defined characteristics, was approved by the China Poultry Genetic Resource Committee in 2015. Thus, to investigate the relationship between the progenitor H line and other poultry breeds, the genetic diversity and population structure of “Yufen 1” H line (YF) were investigated and compared with those of 2 commercial chicken breeds, the ancestor breed Red Jungle Fowls, and 11 Chinese indigenous chicken breeds based on a whole-genome resequencing approach using 8,112,424 SNPs. The genetic diversity of YF was low, and the rate of linkage disequilibrium decay was significantly slower than that of the other Chinese indigenous breeds. In addition, it was shown that the YF population was strongly selected during intensive breeding and that genetic resources have been seriously threatened, which highlights the need to establish a systematic conservation strategy as well as utilization techniques to maintain genetic diversity within YF. Moreover, a principal component analysis, a neighbor-joining tree analysis, a structure analysis, and genetic differentiation indices indicated that YF harbors a distinctive genetic resource with a unique genetic structure separate from that of Chinese indigenous breeds at the genome level. The findings provide a valuable resource and the theoretical basis for the further conservation and utilization of YF.

## 1. Introduction

China is among the countries with the richest poultry genetic resources worldwide [1]. With the domestication of chickens, various types of poultry breeds have been formed through both natural and artificial selection, accumulating considerable genetic and phenotypic variations [2]. In this context, Chinese indigenous poultry breeds have excellent characteristics, such as early sexual maturity, a strong resistance to adversity, a rough feeding resistance, good meat quality, and a low frequency of harmful genes [3]. However, exotic breeds are widely utilized in poultry production as a way to meet market and economic needs. Although the extensive utilization and hybridization of exotic poultry breeds have improved production performance and economic efficiency, they have also negatively impacted the resources of indigenous breeds, especially those in the southeast coastal areas of China, which are on the verge of extinction [4]. In fact, it is estimated that a considerable proportion of indigenous poultry breeds (21.3% worldwide) are endangered due to breeding strategies that are deemed inadequate for the genetic sustainability of indigenous breeds [5]. Therefore, it is particularly urgent to study the genetic diversity and the relationships of indigenous poultry breeds, since this type of assessment can provide the basis for developing more effective resource conservation, development, and utilization strategies.

In recent years, with the reduction in sequencing costs, whole-genome sequencing technology has been applied to several aspects of agricultural practices, including molecular detection and breed conservation [6,7]. Numerous studies have been conducted on genetic diversity and systematic relationships based on various molecular markers at the regional scale and worldwide [8,9]. Sun J et al. [10] assessed the genetic diversity of indigenous poultry from Guangxi provinces using resequencing and found that most of the local populations could be characterized by a higher genetic diversity and lower differentiation compared to commercial chicken breeds. These authors revealed that Guangxi chickens were not significantly affected by recent inbreeding, indicating the effectiveness of breed conservation efforts for indigenous chicken populations in Guangxi. By conducting a population genetic analysis of seven Chinese indigenous chicken breeds in the context of global breeds based on 600 k SNPs, Chen L et al. [11] found that the Dongxiang blue-eggshell and Chongren Partridge (CR) breeds displayed a markedly reduced genetic diversity and a signature of admixture with European commercial breeds, which may help in establishing an efficient conservation program.

In total, 115 different chicken breeds are considered indigenous to China, which was shown to possess great advantages for genetic breeding. However, breeding methods in many parts of China still require considerable development, and several excellent poultry breeds have not yet been efficiently utilized. The matching line of the “Yufen 1” layer was authorized by the National Commission on Livestock and Poultry Genetic Resource in 2015. A closed breeding method was used to develop line H by crossing barred plumaged-original Gushi chicken (GS) with an egg-laying brown-shelled Babcock B-380 grandparent line C. As the progenitor, line H is characterized by early sexual maturity, a fast plumage line, a high egg production, and a Columbian feather pattern [12]. However, knowledge on the genomic structure of the “Yufen 1” H line chicken (YF) is still scant.

Herein, whole-genome sequencing (WGS) data were obtained from ten YF chickens from the poultry germplasm resource farm of Henan Agricultural University. By combining the published whole-genome resequencing data of 2 commercial chicken breeds, 11 Chinese indigenous chicken breeds, and 1 wild chicken breed, the genetic structure of YF representatives was thoroughly evaluated using genetic diversity and population structure analyses. The results discussed constitute scientific and effective data for the development and conservation of YF genetic resources.

## 2. Materials and Methods

### 2.1. Sample Collection and WGS Analysis

A total of 154 individuals from 15 chicken breeds were included in the present study (Supplementary Table S1). Ten blood samples of YF chickens (half males and females, unrelated per breed) were collected from the poultry germplasm resource farm of Henan Agricultural University, China. Genomic DNA was extracted from chicken blood using a TianGen DNA Kit. Paired-end libraries with a ~500 bp insert size were constructed and then subjected to sequencing using the BGISEQ-500 platform to generate 150 bp paired-end reads (BGI Genomics Co., Ltd., Cambridge, MA, USA). The genome resequencing data of the remaining 14 breeds were obtained from previously published data [13,14,15]; the breeds include Huanglang (HL), Huaixiang (HX), Huaibei-Ma (HBM), Hetian (HT), Huxu (HUXU), Jianghan (JH), Ningdu (ND), Wenchang (WC), Wuhua (WH), Yao (YAO), Gushi (GS), Red Jungle Fowl (RJF), White Leghorn (WLH), and Rhode Island Red (RIR). The accession numbers are PRJNA597842, PRJCA004227, PRJNA482210, and PRJEB30270.

### 2.2. Read Mapping and SNP Calling

Clean reads were aligned to the Gallus GRCg6a reference genome using the BWA-MEM alignment algorithm built into BWA (v0.7.17) [16]. Subsequent quality control processes were performed using the SortSam and MarkDuplicates in Picard Tools (v.1.56) [16], followed by variant calling, merging, and filtering using the Genome Analysis Toolkit (GATK v4.1.7.0) [17,18]. Filtering was conducted considering the criteria of QD < 2.0 || MQ < 40.0 || FS > 60.0 || SOR > 3.0 || MQRankSum < −12.5, thereby excluding reads with segregation distortions or sequencing errors. In addition, SNPs were retained using Vcftools (v0.1.16) [19] for subsequent analyses according to the following criteria: a minor allele frequency > 0.1 and a maximum miss rate > 0.8. The Ensemble genome database and SNPEff (v4.1) [20] programs were used to obtain information about SNP annotation. Additionally, the R package CMplot was used to visualize the distribution of the SNPs across the chromosome.

### 2.3. Genomic Diversity Analysis

To determine the genetic diversity among all chicken populations, the average minor allele frequency (MAF), observed heterozygosity (Ho), expected heterozygosity (He), and runs of homozygosity (ROHs) were estimated using PLINK (v1.90). The parameters for the ROH analysis were as follows: a sliding window of 50 SNP slides along the chromosome to estimate homozygosity; each sliding window allowed no more than 1 heterozygote, with no more than 5 missing SNPs, a minimum length of ROH of 100 kb, a minimum density of 1 SNP/50 kb, and a maximum gap between consecutive SNPs of 1000 kb. To estimate individual genomic inbreeding coefficients (FROH) using the ROH data, the length of the genome covered by ROH was divided by the total chicken autosomal genome length covered by SNPs (960,796.788 kb in the present study) [21]. The average genome-wide nucleotide polymorphisms (π) and genetic differentiation (FST) were calculated with a 10 kb sliding window and 5 kb stepwise increments using Vcftools (v0.1.16) [22]. GONE [23] was used to estimate the current generation’s effective population size for all populations. Studies have reported that the total linkage map of the chicken genome ranges from 2600 to 3800 cM [24], and the total genome size is approximately 1100 Mb [25]. A genetic distance of approximately 3 cm is equal to a physical distance of 1 Mb; hence, we used the parameter of 1 Mb = 3 cM for estimation. A linkage disequilibrium (LD) decay analysis was performed using PopLDdecay (v3.41) [26].

### 2.4. Population Genetic Structure and Gene Flow Analysis

Prior to performing a population genetic analysis, the SNPs were identified using LD-based pruned data with PLINK (v1.90), and the parameter was indep—pairwise 25 5 0.2. Based on the pruned SNP data, a principal component analysis (PCA) was conducted using PLINK (v1.90) to explore the genomic repartition in 15 chicken breeds. A neighbor-joining (NJ) tree of the studied populations was constructed based on pairwise distances using PHYLIP (v3.697) [27] and MEGA (v7.0) [28]. The population structure was inferred by applying the model-based clustering algorithm implemented in ADMIXTURE (v1.3.0) software [29]. TreeMix software (v1.13) [30] was used to establish a maximum likelihood tree for a gene flow analysis of the 15 chicken breeds with RJF as the outgroup. Migration events from 1 to 6 were set, and the corresponding residual matrix was generated using the options”-k 500” and “-noss”.

## 3. Results

### 3.1. SNP Calling and Annotation

The samples were sequenced at a genome coverage depth from 5.17× to 32.63×. After quality control and the filtering of unqualified SNPs, a total of 8,112,424 autosomal SNPs were obtained for statistical analyses. After annotating the obtained SNPs, most of the variations were found in introns (29.84%), intergenic regions (10.18%), and transcripts (40.41%) (Supplementary Table S2). A density distribution analysis of the SNPs on each chromosome revealed that SNPs were more frequently distributed on large chromosomes and less frequently distributed on small chromosomes, and the SNP distribution was positively correlated with chromosome length (Supplementary Figure S1). In general, the SNPs appeared to be evenly distributed on each chromosome, except for uneven distribution at the telomeres of some chromosomes.

### 3.2. Genetic Diversity and LD Analysis

The genetic diversity parameters are shown in Table 1. Overall, the genetic diversity indices of YF (Ho = 0.2766, He = 0.2630, MAF = 0.1932, π = 0.0024, and Ne = 70) were between those of commercial and indigenous breeds, and the indigenous breeds showed a higher genetic diversity than the commercial chicken breeds. The genetic diversity indices of RJF were lower (Ho = 0.2724, He = 0.2903, MAF = 0.2155, π = 0.0026, and Ne = 43). The highest average FROH was found in WLH (0.4427) and YF (0.3273), whereas the lowest FROH was identified in HL (0.0284). The overall level of species diversity can be determined by comparing the degree of LD decay among populations. Thus, an LD decay analysis showed that WLH had the slowest LD decay rate, followed by RIR, YF, and the other Chinese indigenous breeds. Conspicuously, the decay rate of YF was significantly slower than that of the Chinese indigenous chicken breeds and RJF (Supplementary Figure S2). To obtain greater insights into the genetic relationship between YF and the other chicken breeds, relatedness was investigated by calculating pairwise FST values (Table 2). It was revealed that the FST values of YF and the Chinese indigenous chicken breeds ranged from 0.1339 (JH) to 0.1472 (ND) with moderate genetic differentiation. In contrast, the FST values between YF and the commercial chicken breeds ranged from 0.2066 (RIR) to 0.2636 (WLH), showing a high genetic differentiation. Within the Chinese indigenous chicken populations, the lowest FST values (0.0056) were found between YAO and HX, suggesting that these chicken breeds have a considerable shared genomic background.

### 3.3. Population Genetic Structure and Gene Flow Analysis

**Figure 1:**
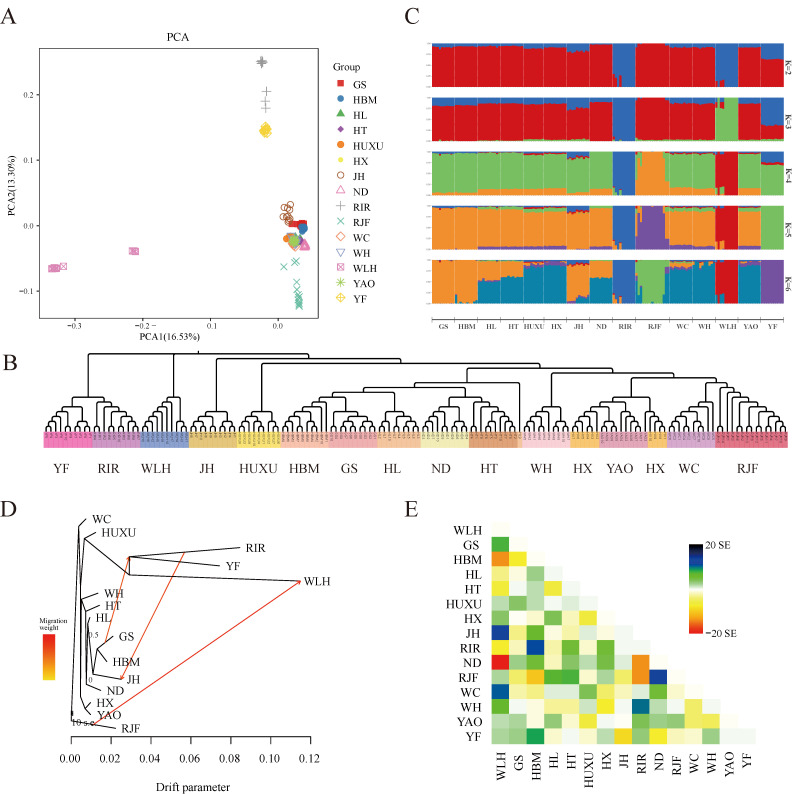
Population structure and gene flow analysis of all chicken individuals. (**A**) First and second principal components from a principal component analysis of all populations; (**B**) neighbor-joining tree for all individual chickens. (**C**) ADMIXTURE analysis for K = 2 up to 6. The same color indicates groups with the same ancestry. (**D**) Maximum likelihood tree with 3 migration events. Migration events are shown as colored arrows, shaded according to their weight. Horizontal branch lengths are proportional to the amount of genetic drift that has occurred at each branch. The scale bar shows 10 times the average standard error of the entries in the sample covariance matrix; (**E**) residual fit from the maximum likelihood tree in (**D**).

PCA showed that the contribution rates of the first two principal components (PCs) to explain the variance in the data were 16.53% and 13.30%, respectively (Figure 1A). PC1 isolated WLH from all the other chicken breeds, whereas PC2 mainly contributed to the separation of YF and RIR from the other indigenous chicken breeds, WLH and RJF. The PCA results were mainly consistent with those obtained for the NJ tree constructed based on whole-genome polymorphic SNPs (Figure 1B). Of note, the NJ tree revealed the formation of 3 genetic clusters: cluster 1 comprised WLH, cluster 2 comprised YF and RIR, and cluster 3 comprised the remaining 11 Chinese indigenous chicken breeds and RJF. Furthermore, in order to determine the historical admixture patterns of the chicken populations, an ADMIXTURE analysis was conducted with K values ranging from 2 to 15. At K = 2, a genetic divergence first occurred between the commercial and noncommercial chicken breeds. When K was increased, WLH (K = 3), RIR (K = 3), RJF (K = 4), and YF (K = 5) were progressively assigned to a distinct cluster (Figure 1C). When K = 5, the cross-validation error reached the lowest value (Supplementary Figure S3 and Supplementary Table S3). To further investigate the historical split and admixture in the studied chicken breeds, a TreeMix analysis was conducted to infer the maximum likelihood population (ML) tree and potential migration events for the chicken breeds (Figure 1D,E). It was found that the inferred migration edges under the assumption of three migration events returned the lowest residuals (Supplementary Figure S4), resulting in a ML tree that best fit the data obtained in the present study. The ML tree explained 80.0% of the variance when considering three migrations. Based on this ML tree, YF and RIR were nonetheless found in a large clade. Taken together, the results from the gene flow analysis suggest that the occurrence of three migration events were as follows: RJF –> WLH, RIR –> JH, and GS –> RIR and YF.

## 4. Discussion

An analysis of genetic diversity and population structure using genetic data can improve or drive animal selection toward maintaining genetic diversity [31]. In this study, genetic diversity, population structure, and genetic differentiation were investigated in commercial chicken breeds, Chinese indigenous breeds, RJF, and YF populations based on whole-genome resequencing data. Furthermore, the genetic distinctiveness of YF chickens was described, which provides useful information for maintaining biodiversity [32,33].

The genetic diversity indices (Ho, He, MAF, π, and Ne) of the two commercial chicken breeds (WLH and RIR) were the lowest compared with those of the indigenous chicken breeds, indicating a lower genetic diversity among the commercial breeds. In addition, the slowest rate of LD decay was found among the two commercial chicken breeds, which could be explained by the fact that both breeds experienced intensive selection for egg production. This hypothesis can be also confirmed by the high FROH values, indicating that these two breeds underwent inbreeding to a considerably large extent [11]. Compared with the other indigenous breeds, YF had lower genetic diversity indices (Ho, He, MAF, π, and Ne), a slower rate of LD decay, and a higher degree of inbreeding. These findings are consistent with the current breeding process of YF. Line H was bred for at least twelve generations and underwent fierce selection and purification, aiming to improve individual growth, reproductive performance, and appearance. Moreover, it was observed that inbreeding occurred among YF populations; thus, the genetic diversity in YF was relatively low. These results highlight the urgent need to preserve the biodiversity in YF, and special attention should be given to the conservation of YF. RJF is the domestic chicken ancestor, and the genetic diversity within RJF should be relatively high [34]. Surprisingly, it was found that the genetic diversity in RJF was even lower than that in most indigenous breeds in this study. The observed low genetic diversity in RJF was mainly due to the RJF sampling scheme, which included closely related individuals from the same subgroup [15], with a high degree of inbreeding (FROH = 0.1696) between the sampled populations. In recent years, increased human activity has led to a decrease in the number and distribution of RJF, which resulted in a decrease in genetic diversity within this breed [35]. Egg harvesting and hunting have been considered the greatest threats to RJF populations [35,36]. Concerning the genetic differentiation indices, the highest values were found between each commercial breed and the Chinese indigenous chickens, and the results are in accordance with those reported in a previous study [1]. In particular, the level of genetic differentiation between YF and the commercial chicken breeds was high, whereas that between YF and the indigenous breeds was moderate. Collectively, these results indicate that YF has unique genetic characteristics, and it is worth further exploring its breed-specific molecular markers.

An effective population size and an appropriate selection and mating strategy are necessary components of species conservation plans. Studies have shown that the appropriate effective population size not only maintains population fitness but also facilitates species monitoring and management [37]. Indeed, through suitable mating plans, specific genetic conservation strategies might be applied to animals with the goal of increasing genetic variability and controlling inbreeding [38]. In a previous study, the genomic diversity among populations of three Chinese indigenous chicken breeds was assessed based on genome-wide SNPs, which revealed that the genetic diversity of conserved populations in which random mating prevails was high, and the inbreeding coefficient was below 0.1 [39]. This result indicated that random mating largely avoided inbreeding and can be considered a solid strategy for improving conservation schemes. Considering the current status of genetic diversity, particular attention should be given to YF, as it showed a lower genomic variability and a higher inbreeding coefficient. Therefore, an elaborate conservation program should be established for YF to avoid further inbreeding and to maintain its genetic diversity. In addition, strategies for building and utilizing genomic resource banks for conservation breeding can also greatly protect the genetic diversity of species [40].

Furthermore, the population structure and historical admixture patterns were assessed for the 15 different chicken breeds. Based on PCA and an NJ tree analysis, each individual of YF clustered together, showing a consistent genetic relationship. Interestingly, the ML tree of the gene flow analysis showed that YF and RIR clustered in a single clade, which was also confirmed by PCA and the NJ tree analysis, thereby suggesting a possible genetic relationship between these two breeds. We believe that the close genetic relationship between YF and RIR is mainly due to their low genetic diversity and drift, which further emphasizes the urgency and importance of conserving the genetic resources of YF. The migration events not only indicated the genetic contribution of the wild chicken breed RJF to the commercial breed WLH but also suggested gene exchange between the commercial chicken breed RIR and the indigenous breeds GS, YF, and JH. Finally, an ADMIXTURE analysis revealed that, at K = 5, WLH, RIR, RJF, and the other 12 indigenous chicken breeds could be distinguished from one another, and YF was clearly assigned to a separate gene pool. These results confirm the reliability of the classification of YF as a separate breed.

## 5. Conclusions

In conclusion, the genetic diversity and population structure of YF, two commercial chicken breeds, indigenous breeds, and the ancestor breed RJF were comprehensively studied based on genome-wide SNPs. The results show that the genetic diversity of YF was low, which may have undergone intensive selective pressure. Additionally, this study demonstrated at the whole-genome level that YF is a precious genetic resource and helped us to understand the genetic structure of YF. Based on the data described herein, conservation strategies should be implemented to maintain genetic diversity in YF, and the potential of this genetic resource should be further explored. In fact, we can control inbreeding through appropriate mating programs and develop in situ and off-site conservation programs to safeguard genetic variability and achieve specific genetic conservation plans for this breed. Moreover, the present data provide useful information about the genomics of Chinese and Western poultry breeds, which will likely assist in the development of a national project for the conservation and utilization of these breeds.

## Figures and Tables

**Table 1:** Genetic diversity indices.

Breeds	N ^1^	Breed Assignment	Ho ^2^	He ^3^	FROH ^4^	Π ^5^	Ne ^6^	MAF ^7^
WLH ^8^	10	Commercial breeds (Italy)	0.1332	0.1757	0.4427	0.0016	11	0.1279
RIR ^9^	10	Commercial breeds (America)	0.1915	0.2128	0.3096	0.0019	59	0.1582
GS ^10^	10	Native breeds (Henan province, China)	0.3129	0.3206	0.0793	0.0028	120	0.2372
HBM ^11^	10	Native breeds (Anhui province, China)	0.2992	0.3056	0.0469	0.0027	167	0.2273
HL ^12^	10	Native breeds (Hunan province, China)	0.3042	0.3174	0.0284	0.0028	497	0.2353
HT ^13^	10	Native breeds (Fujian province, China)	0.3019	0.3095	0.0472	0.0028	306	0.2301
HUXU ^14^	9	Native breeds (Guangdong province, China)	0.2794	0.3072	0.0424	0.0027	183	0.2283
HX ^15^	10	Native breeds (Guangdong province, China)	0.2991	0.3133	0.0405	0.0028	262	0.2325
JH ^16^	10	Native breeds (Hubei province, China)	0.2817	0.3064	0.0784	0.0027	52	0.2278
ND ^17^	10	Native breeds (Jiangxi province, China)	0.2891	0.3055	0.0540	0.0027	262	0.2269
WC ^18^	10	Native breeds (Hainan province, China)	0.2936	0.3134	0.0374	0.0028	216	0.2324
WH ^19^	10	Native breeds (Anhui province, China)	0.3033	0.3078	0.0352	0.0027	52	0.2288
YAO ^20^	10	Native breeds (Guangxi province, China)	0.3026	0.3136	0.0422	0.0028	264	0.2328
RJF ^21^	15	Wild breeds (Thailand)	0.2724	0.2903	0.1696	0.0026	43	0.2155
YF ^22^	10	Native breeds (Henan province, China)	0.2766	0.2630	0.3273	0.0024	70	0.1932

^1^ N = the number of animals per breed, ^2^ Ho = observed heterozygosity, ^3^ He = expected heterozygosity, ^4^ FROH = inbreeding coefficients, ^5^ π = nucleotide polymorphisms, ^6^ Ne = effective population size, ^7^ MAF = minor allele frequency, ^8^ WLH = White Leghorn, ^9^ RIR = Rhode Island Red, ^10^ GS = Gushi, ^11^ HBM = Huaibei-Ma, ^12^ HL = Huanglang, ^13^ HT = Hetian, ^14^ HUXU = Huxu, ^15^ HX = Huaixiang, ^16^ JH = Jianghan, ^17^ ND = Ningdu, ^18^ WC = Wenchang, ^19^ WH = Wuhua, ^20^ YAO = Yao, ^21^ RJF = Red Jungle Fowl, ^22^ YF = “Yufen 1” H line.

**Table 2:** Genetic differentiation index between populations.

Breeds	WLH ^1^	RIR ^2^	GS ^3^	HBM ^4^	HL ^5^	HT ^6^	HUXU ^7^	HX ^8^	JH ^9^	ND ^10^	WC ^11^	WH ^12^	YAO ^13^	RJF ^14^	YF ^15^
WLH ^1^	0	0.3006	0.2232	0.2239	0.2039	0.2123	0.2043	0.2010	0.2079	0.2164	0.1989	0.2092	0.2020	0.2127	0.2636
RIR ^2^		0	0.1648	0.1648	0.1580	0.1650	0.1619	0.1584	0.1480	0.1712	0.1593	0.1654	0.1589	0.1947	0.2066
GS ^3^			0	0.0278	0.0272	0.0429	0.0460	0.0437	0.0405	0.0424	0.0428	0.0492	0.0437	0.0947	0.1404
HBM ^4^				0	0.0193	0.0365	0.0405	0.0386	0.0333	0.0350	0.0371	0.0438	0.0390	0.0921	0.1411
HL ^5^					0	0.0166	0.0193	0.0149	0.0262	0.0151	0.0135	0.0240	0.0163	0.0683	0.1347
HT ^6^						0	0.0307	0.0278	0.0417	0.0254	0.0244	0.0355	0.0288	0.0761	0.1427
HUXU ^7^							0	0.0205	0.0398	0.0320	0.0170	0.0293	0.0221	0.0746	0.1372
HX ^8^								0	0.0390	0.0274	0.0166	0.0272	0.0056	0.0696	0.1368
JH ^9^									0	0.0410	0.0375	0.0452	0.0393	0.0903	0.1339
ND ^10^										0	0.0253	0.0349	0.0291	0.0757	0.1472
WC ^11^											0	0.0249	0.0179	0.0650	0.1378
WH ^12^												0	0.0280	0.0781	0.1432
YAO ^13^													0	0.0706	0.1372
RJF ^14^														0	0.1780
YF ^15^															0

^1^ WLH = White Leghorn, ^2^ RIR = Rhode Island Red, ^3^ GS = Gushi, ^4^ HBM = Huaibei-Ma, ^5^ HL = Huanglang, ^6^ HT = Hetian, ^7^ HUXU = Huxu, ^8^ HX = Huaixiang, ^9^ JH = Jianghan, ^10^ ND = Ningdu, ^11^ WC = Wenchang, ^12^ WH = Wuhua, ^13^ YAO = Yao, ^14^ RJF = Red Jungle Fowl, ^15^ YF = “Yufen 1” H line.

## Data Availability

All the sequence data and SNP data generated in this study were deposited in the National Genomics Data Center (https://bigd.big.ac.cn (accessed on 18 October 2022)) with the accession codes PRJCA012580 and PRJCA013041.

## Supplementary Materials

The following supporting information can be downloaded at https://www.mdpi.com/article/10.3390/life13030793/s1, Supplementary Figure S1: The location of SNPs along chromosomes. The *x*-axis represents the chromosome position (Mb), and the *y*-axis denotes the chromosomes. The number of SNPs present in each 1 Mb genome block is expressed via colors as indicated in the key; Supplementary Figure S2: LD decay in different populations within 300 kb; Supplementary Figure S3: The cross-validation error for K = 2 to 15; Supplementary Figure S4: Residual matrix of each migration event scenario (from 1 to 6 migration events, corresponding to A–F, respectively); the ML tree with 3 migration edges showing the smallest residuals; Supplementary Table S1: List of chicken breeds used for analyses; Supplementary Table S2: Distribution of variants in functional regions of the chicken genome; Supplementary Table S3: The cross-validation error values for K = 2 to 15.
