# Supplementary material for: Population Structure and Genetic Diversity Analysis of “Yufen 1” H Line Chickens Using Whole-Genome Resequencing
Source: Life (Basel). 2023 Mar 15;13(3):793. doi: 10.3390/life13030793 (PMC10059704; doi:10.3390/life13030793)
Supplement: Supplementary file 1 [file life-13-00793-s001.zip › Supplementary Figure S4.pdf]

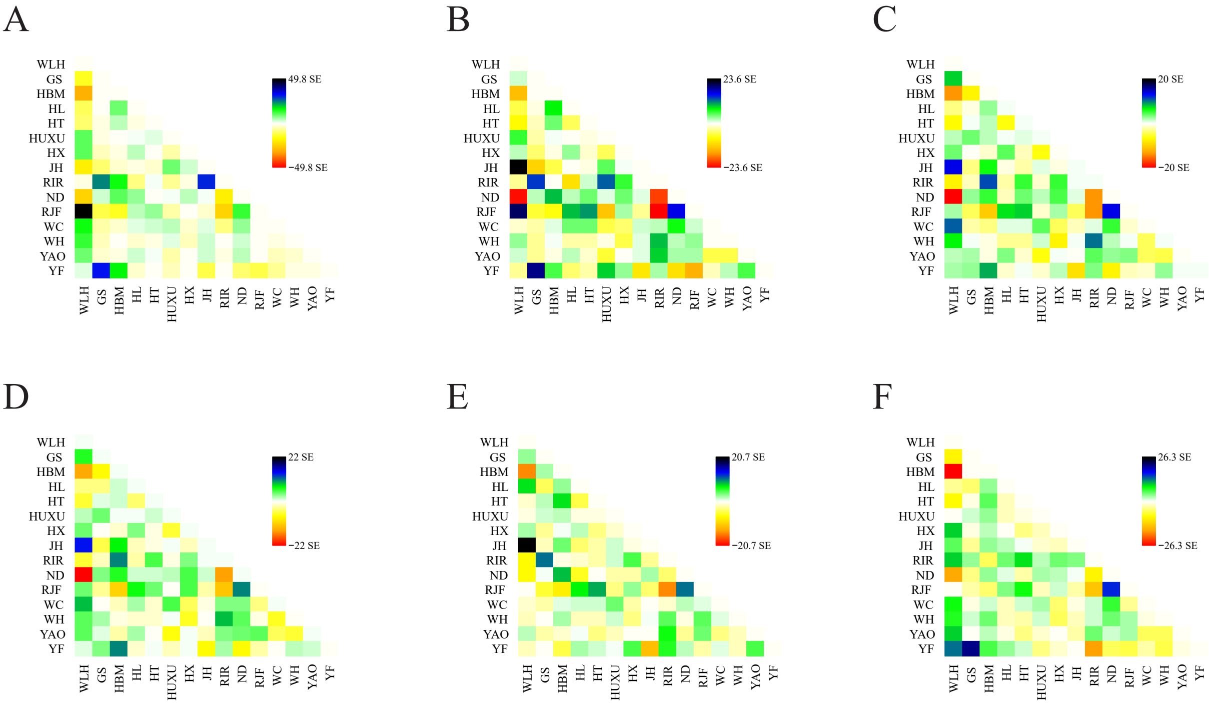
A
B
C
D
E
F
WLH
GS
HBM
HL
HT
HUXU
HX
JH
RIR
ND
RJF
WC
WH
YAO
YF
49.8 SE
−49.8 SE
23.6 SE
−23.6 SE
20 SE
−20 SE
22 SE
−22 SE
20.7 SE
−20.7 SE
26.3 SE
−26.3 SE
