# Supplementary figures and images for: Population Structure and Genetic Diversity Analysis of “Yufen 1” H Line Chickens Using Whole-Genome Resequencing
Source: Life (Basel). 2023 Mar 15;13(3):793. doi: 10.3390/life13030793 (PMC10059704; doi:10.3390/life13030793)

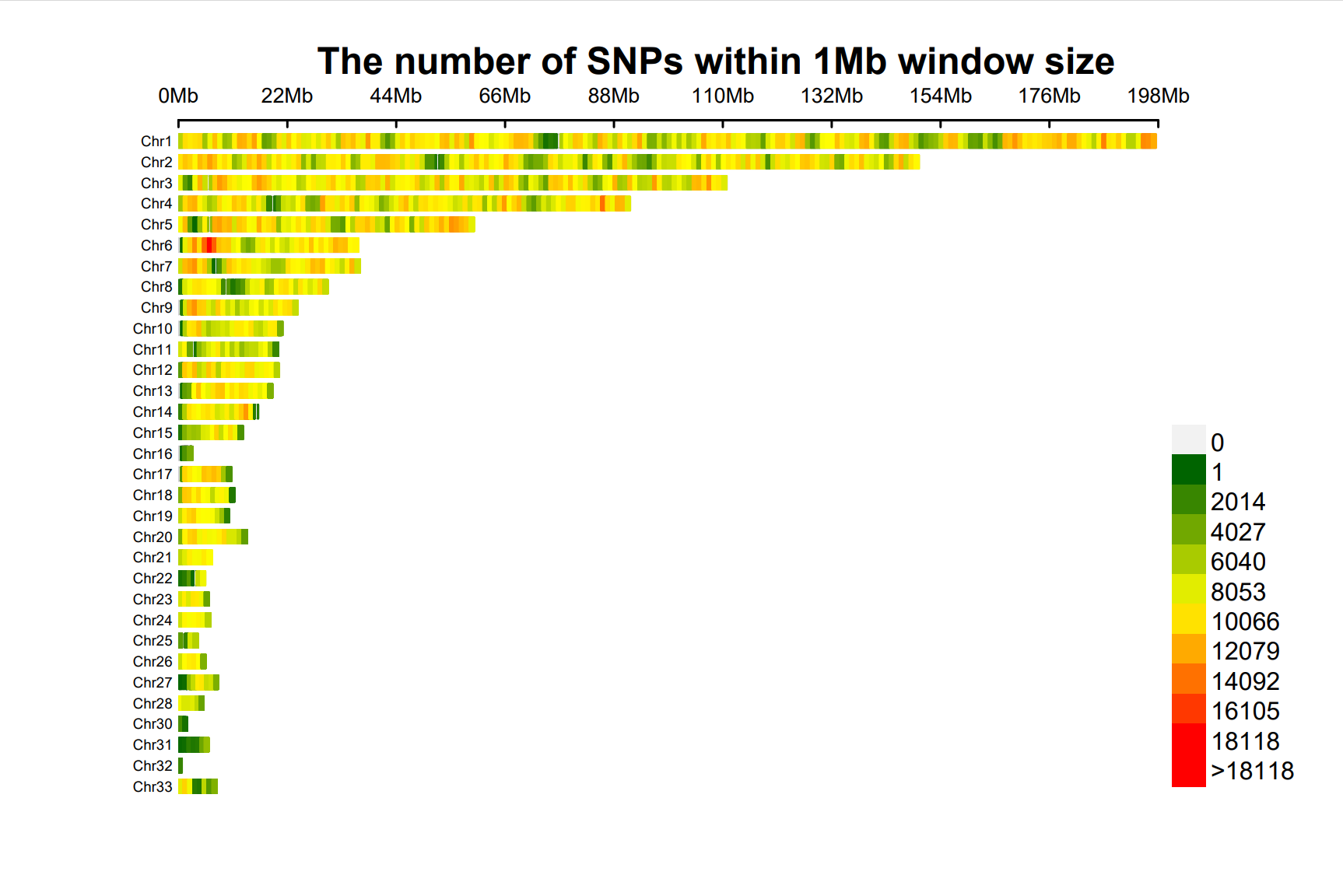

Supplement: Supplementary file 1 [file life-13-00793-s001.zip › Supplementary Figure S1.png]

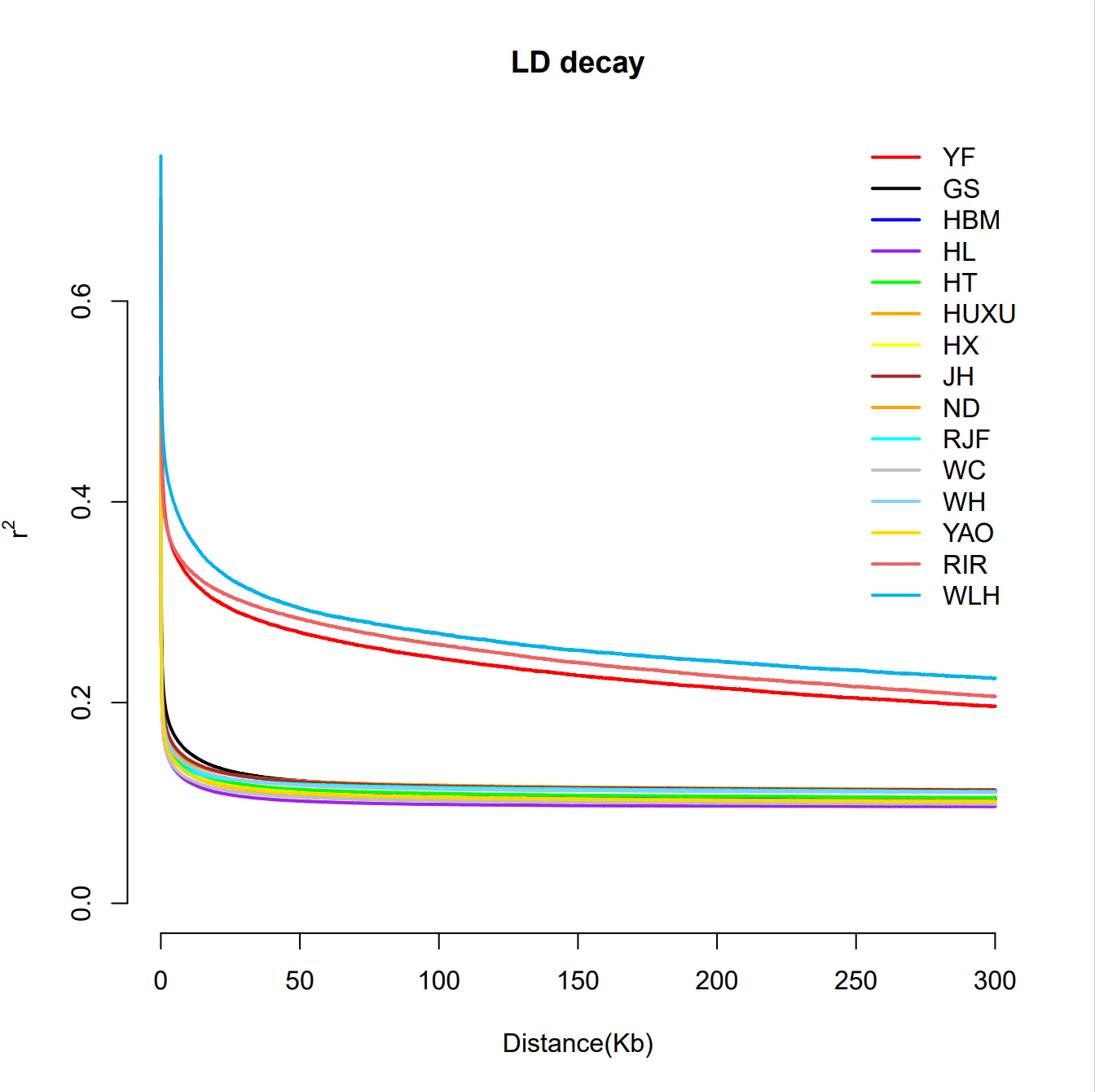

Supplement: Supplementary file 1 [file life-13-00793-s001.zip › Supplementary Figure S2.png]

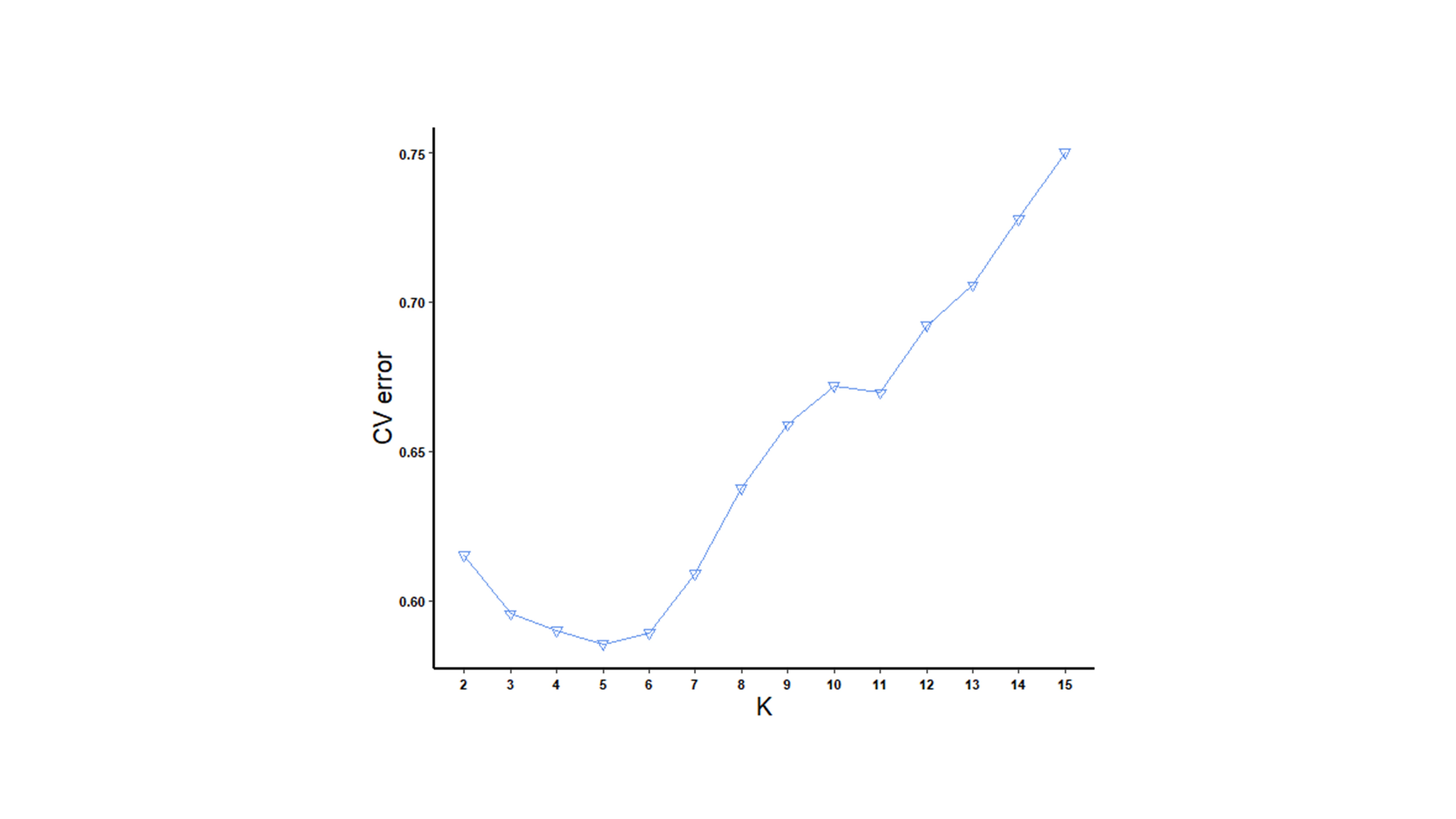

Supplement: Supplementary file 1 [file life-13-00793-s001.zip › Supplementary Figure S3.JPG]
